# Intrinsic electrical properties of mammalian neurons and CNS function: a historical perspective

**DOI:** 10.3389/fncel.2014.00320

**Published:** 2014-11-04

**Authors:** Rodolfo R. Llinás

**Affiliations:** Department of Neuroscience and Physiology, New York University School of MedicineNew York, NY, USA

**Keywords:** oscillations, voltage-gated ion channels, oscillatory phase reset, mammalian neurons, oscillatory resonance

## Abstract

This brief review summarizes work done in mammalian neuroscience concerning the intrinsic electrophysiological properties of four neuronal types; Cerebellar Purkinje cells, inferior olivary cells, thalamic cells, and some cortical interneurons. It is a personal perspective addressing an interesting time in neuroscience when the reflex view of brain function, as the paradigm to understand global neuroscience, began to be modified toward one in which sensory input modulates rather than dictates brain function. The perspective of the paper is not a comprehensive description of the intrinsic electrical properties of all nerve cells but rather addresses a set of cell types that provide indicative examples of mechanisms that modulate brain function.

## Introduction

That the function of the nervous system is ultimately to be defined as the product of interacting networks woven by nerve cells has been the central dogma of neuroscience for almost a century. Fundamental to this view has been the realization that nerve cells are truly individual elements. Indeed, while the variety of forms that nerve cells may display was described in elegant detail by the work of brilliant morphologists of the turn of the century, their most significant contribution was the proposal of the neuron doctrine (cf. Ramón y Cajal, 1904).

On the other hand, from a physiological point of view the neuron doctrine was considered for a long time to signify a unity of excitability, where the variance among the different neurons related to their shape and connectivity, but not to their individual electrophysiological properties. Thus, following the discovery of excitatory and inhibitory synaptic potentials, it was assumed that the necessary and sufficient functional coinage for the expression of functionality in nerve nets had been defined. Over the past 30 years, however, another fundamental issue has arisen with respect to the physiological properties of nerve cells—that of their intrinsic electroresponsive properties. This concept may be stated simply: “Neuronal types are not interchangeable.” That is, a neuron of a given type (e.g., a thalamic cell) cannot be functionally replaced by one of another type (e.g., an inferior olivary cell), even if their synaptic connectivity and the type of neurotransmitter outputs are identical. (The difference is that the intrinsic electrophysiological properties of thalamic cells are extraordinarily different from those of inferior olivary neurons).

This being the case, the intrinsic electrophysiological signature of nerve cells becomes a central theme in neuronal function. Indeed, when such elements interconnect, the dynamics of the resulting neuronal networks are governed not only by the flow of synaptic current, but also by the intrinsic properties of the neurons partaking in such circuits. Likewise, the electrical activity observed in a network is not only related to the excitatory and inhibitory interactions among neurons but also to their inherent or intrinsic electrical activity (Llinás and Hess, 1976; Llinás, 1988).

The term “intrinsic electrical properties” has been used to encompass both passive and active membrane characteristics (for example, see van Lunteren and Dick, 1992). In this review it is used in a more restricted sense to designate those active membrane properties that endow a cell with the ability to shape incoming stimuli and indeed to fire or maintain subthreshold oscillations in the absence of synaptic input. That is, these cells are capable of more than the classical input-output relationship of increasing their firing frequency with stimulus strength or action of neuromodulators (see Binder et al., 1993, for motoneurons). Due to the presence of bursting, neurons with such usual properties were first recognized in systems concerned with rhythmic activity such as breathing, swallowing and chewing. Indeed, the rhythmic firing of hypoglossal neurons was reported as early as 1973 (Lund and Dellow, 1973). However, the contribution of intrinsic electrical properties of hypoglossal motoneurons to such periodicity was not recognized, but rather thought to arise from the action of excitatory and inhibitory synaptic inputs, the presence of gap junctions, and input from a central pattern generator. It was not until much later that the role of the intrinsic electrical properties of the neurons themselves was recognized (see Ramirez and Richter, 1996, for a review of respiratory neurons). In fact, when hippocampal neurons were observed to fire spontaneously when inhibitory input was blocked, the authors concluded “It remains to be determined whether neural properties and connectivity found to be important in this hippocampal rhythm may also play a role in the generation of other rhythmic activities in the mammalian CNS” (Wong et al., 1984). Such spontaneous rhythmicity had been reported for the inferior olive (IO) *in vivo* as early as 1968 (Armstrong et al., 1968). That they were indeed intrinsic to the IO cell was shown in 1986 (Llinás and Yarom, 1986).

Below, some examples are given from our work that illustrate different intrinsic electrical properties in mammalian central neurons. Very surprising, the role of intrinsic activity in the generation of motricity, initially proposed by Graham-Brown (1911), was forgotten for more than halve a century.

## Intrinsic electrical properties of specific cell types

### Cerebellar purkinje cells

The question of intrinsic electroresponsive properties in vertebrate CNS neurons was first encountered in the detailed study of cerebellar Purkinje cells (Llinás and Hess, 1976; Llinás and Sugimori, 1980a,b). These studies demonstrated that Purkinje cells have intricate firing properties and that the dendritic and somatic membranes each have markedly different voltage-dependent conductances that are supported by different types of ionic channels, which combine to give these cells their unique firing signature. At the somatic level, in addition to the Na^+^ and K^+^ conductances that generate the fast action potential, a voltage-dependent, persistent, or very slowly inactivating Na^+^ conductance [g_Na(p)_ (p for persistent)] was also initially encountered in these neurons (Llinás and Sugimori, 1980a). This latter conductance generates a slow, tetrodotoxin (TTX)-sensitive depolarizing response, which, once activated generates prolonged plateau potentials that may last for tens to hundreds of milliseconds (presently known as an “up state”).

**Figure 1:**
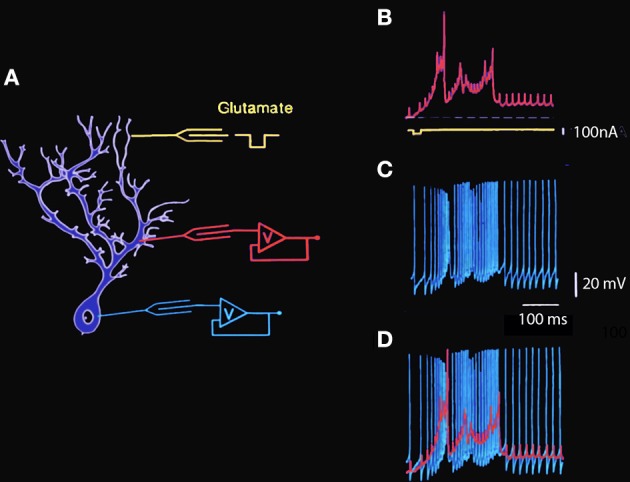
**Simultaneous intracellular recording from Guinea pig cerebellar Purkinje cell dendrite and soma *in vitro*. (A)** Diagram of intracellular recording sites at somatic and dendritic levels and the location of the extracellular glutamic acid iontophoretic application site. **(B)** Intradendritic recording. The large amplitude wide action potentials are Ca-dependent while the smaller fast action potentials represent the passive invasion of the somatic action potentials into the dendritic tree. Note the presence of a sustained plateau depolarization at the dendritic level following the spiking phase of the dendritic response. **(C)** Simultaneous intrasomatic recording showing fast somatic Na-dependent action potentials. Note that each of the large somatic spikes is seen at dendritic level with a short delay and that the calcium dependent dendritic spikes generate high frequency spiking as somatic level. **(D)** Superposition of dendritic (red) and somatic (blue) spikes to illustrate the temporal relationship between somatic and dendritic spikes and plateau amplitudes (Llinás and Sugimori, 1980a,b. This example is unpublished).

This gNa(p) has also been described in cortical (Connors et al., 1982; Stafstrom et al., 1982) and thalamic (Jahnsen and Llinás, 1984a,b) neurons. The dendrites of Purkinje cells, by contrast, do not support voltage-gated Na^+^ conductances, but rather voltage-gated Ca^2+^ conductances that generate dendritic Ca^2+^-dependent spikes and/or plateau potentials (Llinás and Hess, 1976; Llinás and Sugimori, 1980a,b) and are supported by a calcium channel named the P channel (for Purkinje cell). These different membrane conductances and their distribution over the somato-dendritic plasmalemmal membrane endow Purkinje cells with intricate electroresponsive properties, including intrinsic transmembrane voltage oscillations. Such activity can be evoked by direct current injection or by extracellular iontophoretic application of an excitatory transmitter such as glutamic acid at the dendritic level (Figure 1).

The characteristics of somatic and dendritic electroresponsiveness to dendritic glutamate application have been studied using double impalement of Purkinje cells in cerebellar slices. Recordings made during one such experiment in which glutamic acid was applied to the distal dendritic tree are shown in Figure 1. The schematic to the left shows the approximate location of the dendritic and somatic recording electrodes and the iontophoretic glutamate electrode. The trace in B illustrates the main features of dendritic electroresponsiveness. There are two types of Ca^2+^-dependent responses: maintained all-or-none depolarizing plateau responses and slow-rising spikes. The plateau responses have constant amplitude, may last for hundreds of milliseconds, are accompanied by a large conductance increase, and are usually not seen in the soma. On the other hand, the Ca^2+^-dependent spikes in the dendrites are large and are usually elicited in prolonged bursts (Figure 1B), which influence somatic electroresponsiveness. As shown in Figure 1C, they may be recorded in the soma as slow changes in the membrane potential that trigger increases in the firing frequency of the fast, sodium-dependent somatic action potentials. In turn, the somatic action potentials can be observed in the dendrites below the mid-dendritic level as small, fast-rising depolarization.

**Figure 2:**
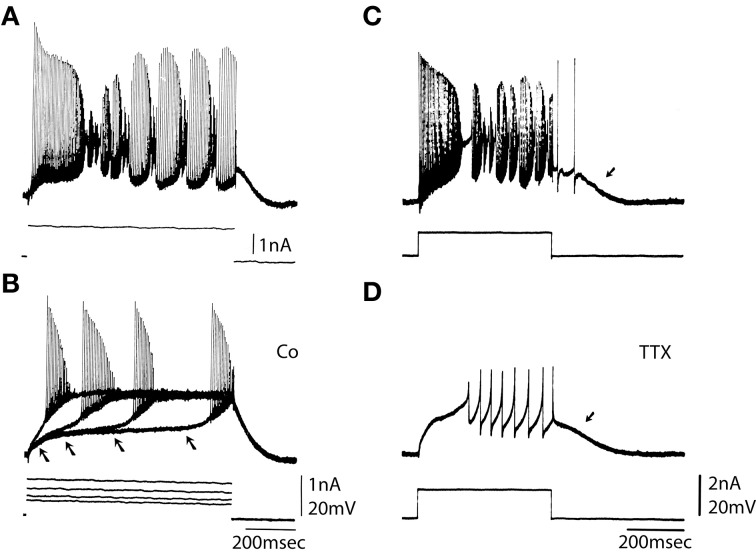
**Ionic basis for Purkinje somatic recordings. (A)** Activity elicited from Purkinje cell soma by direct depolarization. Note fast spikes and underlying slower depolarizations. **(B)** After blocking the calcium conductance by addition of Co to the bath direct depolarization elicited fast spikes. Note that with increased depolarization spike onset moved to the left (arrows), but the plateau level of spike threshold did not change. **(C)** Repetitive response to somatic depolarization. **(D)** Block of sodium channels with TTX reveals underlying slow spikes and afterdepolarization (arrow). (From Llinás and Sugimori, 1980a.).

The ionic basis for Purkinje cell firing was examined by studying the response to depolarizing pulses in the absence of Ca currents and in the absence of Na currents (Figure 2). Addition of Co to the bath blocked the calcium conductance. Direct depolarization elicited fast somatic spikes on a slow depolarizing ramp bringing the membrane to a plateau potential. When the amplitude of the depolarizing pulse was increased the depolarizing ramp occurred earlier (Figure 2B, arrows) without changing the spike threshold or plateau level (Figure 2B). Pharmacological block of the fast sodium channel by addition of TTX to the bath changed the firing pattern as shown in Figure 2D. The fast somatic spikes were blocked while the slow dendritic spike burst and afterdepolarization (Figure 2D, arrow) remained (compare Figure 2C and Figure 2D).

These experiments have provided valuable information relating to the ionic basis of the electrical responsiveness of the soma and dendritic trees and allowed the determination of electrotonic length and some of the active membrane properties of Purkinje cells in general. Yet, they do not provide a direct demonstration of the spatio-temporal distribution of electroresponsiveness over the entire soma dendritic membrane. This requires the use of techniques such as ion-sensitive dyes.

The spatial distribution of ionic channels over the plasmalemmal membrane and the associated compartmentalization of both the physiological and the cell biological properties are critical issues in the characterization of central neuronal function. For example, the precise distribution of specific channels with respect to the locus of synaptic input may address not only electrical integrative properties but also the precision with which different compartments may be addressed biochemically. Indeed, the spatial distribution of second messenger systems activated by [Ca^2+^]_i_ (Hemmings et al., 1986) will be determined by the distribution of calcium channels.

**Figure 3:**
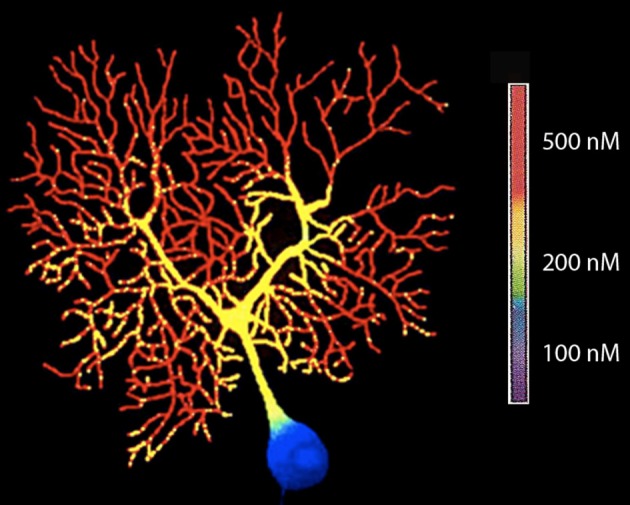
**High-resolution fluorescent image of a dendritic calcium spike in a Purkinje cell filled with fura-2 by microinjection (380-nm excitation)**. (From Tank et al., 1988.).

An early experiment of this type was carried out 26 years ago by Tank et al. (1988). In these experiment Fura II signals were used to determine [Ca^2+^]_i_ in Purkinje cells (Tank et al., 1988). Fura II was injected ionophoretically into the cell and a quantitative evaluation of changes in [Ca^2+^]_i_ was made using the fluorescence ration at 340/380 nM as seen in Figure 3.

The results agreed with the hypotheses of the dendritic segregation of Ca^2+^ conductances that was suggested by early electrophysiological experiments (Llinás and Hess, 1976; Llinás and Sugimori, 1980a,b). They also allow a general mapping of the location of voltage-gated Ca2+ channels as inferred from the specific regions of the neuron, where [Ca^2+^]_i_ demonstrate transients lasting 5–15 ms.

With respect to the functional significance of the results, these findings indicated that the Ca^2+^-dependent plateau potentials are a dendritic boosting mechanism for the synaptic current generated in Purkinje cell dendrites leading to a high-frequency burst of sodium spikes at the soma and axon. This provides a mechanism for spatial and temporal summation of inhibition at the cerebellar nuclear neurons. Other possibilities to be considered relate to role in increased intracellular calcium in the modulation of cell biological mechanisms and the modification of long-term cell biological properties.

**Figure 4:**
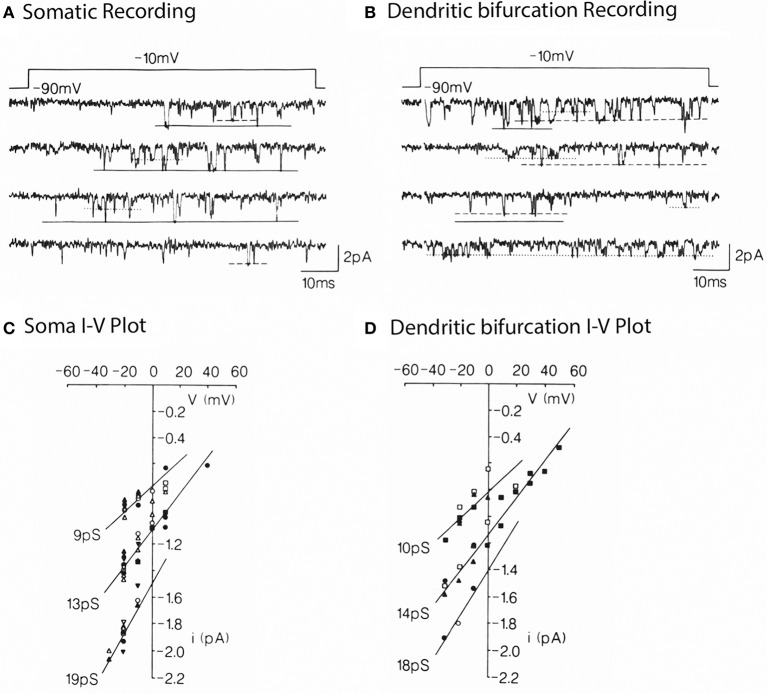
**Multiple conductance of Ca^2+^ channels in the somata and dendrites of cerebellar Purkinje cells. (A)** Single Ca^2+^ channel currents carried by 110 mM Ba^2+^ in a somatic patch, evoked by voltage step jumps (≈70 ms) applied once every 5 s. Three opening levels are indicated by solid, dashed, and dotted lines. **(B)** Currents in a dendritic patch. Same conditions as in **(A)**. Voltage dependence **(C,D)** for the currents levels illustrated in **(A,B)**. Pooled data for 8 somatic and 5 dendritic patches. The indicated conductances are the slope of the lines through the dots (from Usowicz et al., 1992).

The next approach in Purkinje cells was to carry out direct single channel recordings (Figure 4) at both the somatic and dendritic levels (Usowicz et al., 1992). The location of channels on dendrites made it clear that, given the dendritic surface to volume factor relationship, calcium dye imaging would be more effectively implemented at dendritic level. On the other hand, it was also clear that the final calcium concentration change at the cytosolic level would be larger and probably longer lasting at the dendritic than at the somatic level.

In short then, the evidence was clear that Purkinje cells have complex intrinsic properties from the merging of dendritic and somatic conductances giving these cells a unique electrophysiological signature.

### Inferior olivary cells and rebound calcium spikes

**Figure 5:**
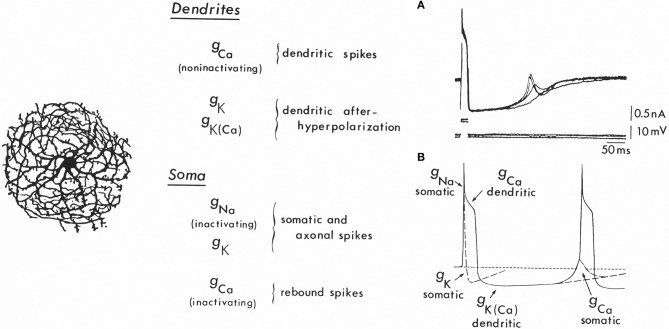
**Ionic conductances and the mechanism for oscillation in inferior olivary cells. Left:** Drawing of an inferior olivary cell by Ramón y Cajal. **Center:** Table giving the distribution of ionic conductances in somatic and dendritic regions. At the soma a set of conductances (g_Na_ and g_k_) generating fast action potentials may be observed. In addition, a strongly inactivated Ca^2+^ conductance is present, which produces rebound spikes, as seen in **(B)** [g_Ca (somatic)_]. Also recorded at the soma is a large Ca^2+^-dependent dendritic spike [g_Ca (dendtritic)_] that generates the afterdepolarization and the powerful, long-lasting afterhypolarization, which is produced by a Ca^2+^-dependent K+ conductance [g_K(Ca)_]. In addition, a voltage-dependent K^+^ conductance (g_K_) seems to be present in the dendrites. **Right A:** Rebound spikes in the inferior olivary neuron (arrow) following blockage of the Na spike with tetrodotoxin (TTX). **Right B:** Summary of the ionic conductances that generate single-cell oscillations in neurons of the inferior olive.

Cells of the inferior olivary nucleus have also been shown to have dendritic and somatic conductances underlying an intrinsic electrophysiological profile. Indeed, *in vitro* experiments using brainstem slices (Llinás and Yarom, 1981, 1986) first demonstrated that IO neurons have a set of voltage-gated ionic conductances that give these cells intrinsic oscillatory properties (Figure 5). Thus, the firing of IO cells is characterized by an initial fast-rising action potential (a somatic sodium spike), which is prolonged to 10–15 ms by an afterdepolarization (a Ca^2+^-dependent dendritic spike).

The abrupt long-lasting afterhyperpolarization (AHP) following the plateau afterdepolarization totally silences the spike-generating activity. This hyperpolarization is typically terminated by a sharp, active rebound response (Figure 5A, arrow), which arises when the membrane potential is negative to the resting level. This rebound response is due to the activation of a somatic Ca^2+^-dependent action potential and results from a second voltage-dependent Ca^2+^ conductance, which is inactive at the resting membrane potential (−65 mV). Membrane hyperpolarization deinactivates this conductance, and, as the membrane potential returns to baseline, a “low threshold” Ca^2+^-dependent spike is generated (Llinás and Yarom, 1981). The rebound potential can be modulated by small changes in the resting membrane potential such that a full Na^+^ spike, which, in turn, can set forth the whole sequence of events once again, is activated. In this way, the cell will fire at a frequency determined largely by the characteristics of the AHP (Figure 5B).

**Figure 6:**
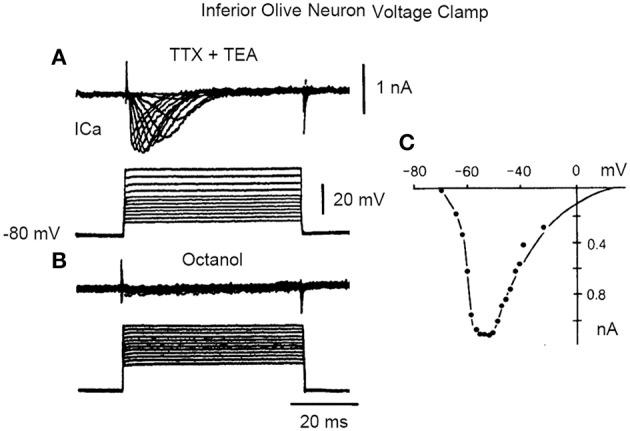
**Inward current in inferior olive cell after block of sodium and potassium currents with TTX and TEA, respectively. (A)** A set of transmembrane square voltage camp steps of increasing amplitude generated a rapidly inactivating, transient, Ca current (Ica). **(B)** This current is blocked by addition of octanol. **(C)** Plot of the current voltage relation in **(A)**. (From Llinás and Yarom in Llinás et al., 1989).

A direct demonstration of time course and amplitude of the “low threshold” transient calcium current [ICA (T)], encountered in this neuron (Llinás and Yarom, 1981) is shown in Figure 6, following a voltage clamp study of IO neuronal calcium currents.

**Figure 7:**
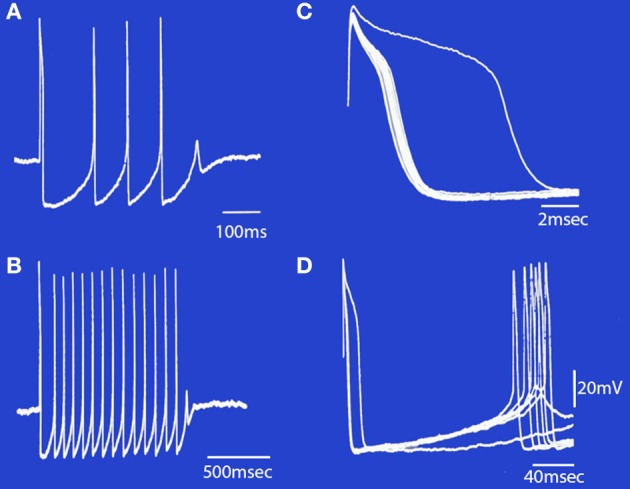
**Spontaneous bursts of spikes recorded intracellularly from an IO neuron displayed at different sweep speeds. (A)** The neuron fired four action potentials and a fifth subthreshold response that corresponds to a subthreshold somatic Ca^2+^-dependent spike. **(B)** A longer burst of spikes is shown at a slower sweep speed. Note that the first interspike interval in the burst was longer than the rest. **(C)** The rising phase of the action potentials in **(B)** are superimposed to illustrate the change in after-depolarization duration during the train. Note that the first action potential (which arises from the resting membrane potential level) has the longest after-depolarization. The other spikes in the train became progressively shorter until failure of spike generation occurred and the burst terminated. **(D)** The same set of records as in **(B)**, showing the duration of the after-hyperpolarization and the rebound somatic Ca^2+^-dependent spikes. (Modified from Llinás and Yarom, 1986).

The low threshold, transient calcium current is a powerful modulator of IO rhythmicity and is responsible for IO membrane potential oscillations. IO neuron oscillations can occur at two distinct frequencies, as determined by examining the firing properties of spontaneous bursts of spikes. A set of such events is shown in Figure 7.

It is evident, given the above, that individual IO cells can oscillate with two main limit cycles, one near 10 Hz (9–12 HZ) and the other near 4 HZ (3–6 HZ). Oscillation at the higher frequency seems to be governed by the resting potential of the neuron. Thus, when the cell is depolarized, its excitability would be dominated by the dendritic conductances and fire near 4 Hz. However, when the cell is hyperpolarized, its output is dependent on somatic conductances and will fire near 10 Hz.

**Figure 8:**
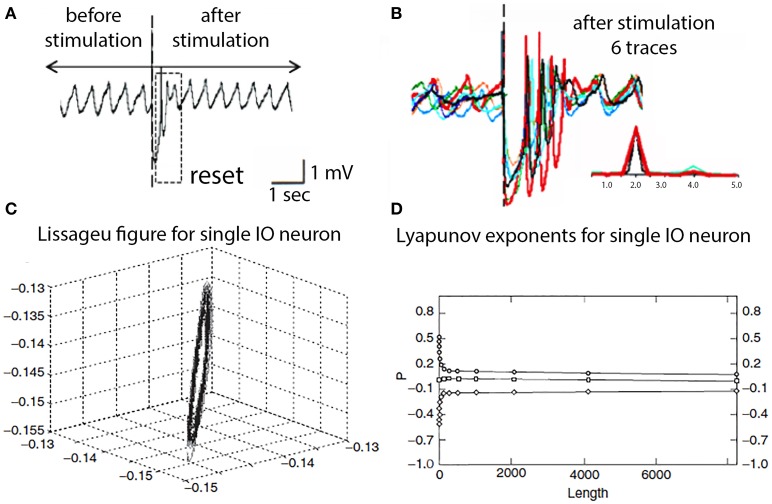
**IO oscillatory properties following spike activation. (A)** One extracellular stimulus briefly interrupted the spontaneous oscillation. **(B)** Superimposition of six traces demonstrating the reset oscillatory phase is the same regardless at which point of the intrinsic oscillation the stimulus was delivered. Inset, power spectra for traces. (Leznik et al., 2002). **(C)** Lissageu figure obtained from the analysis of an IO neuron oscillation. The regularity of the figure shows that the IO attractor has a regular, periodic trajectory. **(D)** Calculated Lyapunov exponents indicative of low-dimensional chaotic dynamics.

Beyond its intrinsic oscillatory behavior, one of the most unexpected and novel properties of the dynamics of IO neurons was their phase-reset ability. Thus, synaptic input large enough to activate action potentials also produces a phase reset of the oscillatory rhythm that is independent of the phase point at which the stimulus arrived. As shown in Figure 8A, a stimulus large enough to generate a spike discharge is immediately followed by a rapid return of the membrane potential oscillatory behavior. If the spike-activating stimulus is repeated, as in Figure 8B, it becomes apparent that the resultant oscillatory phase reset is the same regardless of the moment in time when the stimulus was delivered.

These oscillatory membrane potential properties can also be demonstrated to have interesting dynamic properties. Analysis of the oscillatory dynamics such as shown in Figures 8A,B demonstrated that IO cells have attractor properties as reconstructed from a time series analysis that has a structure close to a limit cycle with a regular periodic trajectory (Makarenko and Llinas, 1998). Average mutual information and false near neighbor methods were calculated and are shown in Figures 8C,D.

To reconstruct the attractor and Lyapunov exponents were derived and the results demonstrate zero, positive and negative exponents values indicating that the system displays low-dimensional chaotic dynamics, that actually explain the phase rest properties of their oscillation, as shown in Figure 8B. The application of this modeling to IO dynamics shown that the subthreshold oscillations support low dimensional chaotic dynamics and that IO electronic coupling leads to rapidly generated complex functional states without increasing the dimensionality of the system (Makarenko and Llinas, 1998).

Thus, in addition to uniform membrane potential oscillatory properties, because of their dynamics IO neurons have the unique ability to reset their oscillatory phase when activated (Leznik et al., 2002; Lefler et al., 2013). This reset property has been found to be functionally very significant as it allows a rapid reset of motricity when dominated by the somatic conductances and fire near 10 Hz—the basic rhythmicity of motor control in vertebrates (Vallbo and Wessberg, 1993; Lang et al., 2006). It has also been shown to be essential in the rapid reorganization of motricity following motor stumbling, even under robotic control (Porras and Llinás, 2014).

At the cerebellar level the functional significance of the oscillatory properties illustrated in Figure 8 is an increased probability of Purkinje cell complex spike activation relating to rapid recovery of motor execution following stumbling, or other unpredicted motor events. Ultimately, then IO oscillatory activity is required for proper motor execution, as demonstrated by the total ataxia that follows T type calcium channels knockout (Choi et al., 2010).

### Thalamic cells

**Figure 9:**
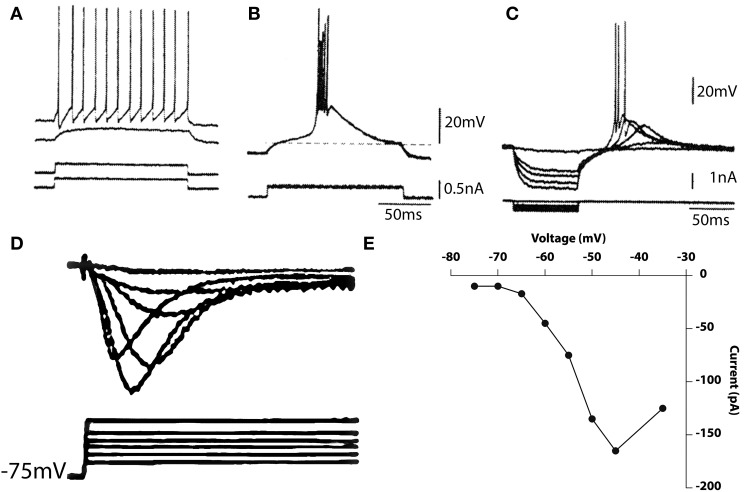
**Electrophysiological properties of thalamic cells recorded *in vitro*. (A,B)** Depolarizing current pulses (bottom traces) elicited no response when delivered from the resting potential, tonic firing when delivered from a depolarized potential **(A)** and a burst response when delivered from a hyperpolarized level **(B)**. **(C)** Rebound response seen after hyperpolarizing pulses. **(D,E)** Calcium currents elicited by membrane depolarization from a hyperpolarized potential **(D)** and current–voltage relationship **(E)**. (Geijo-Barrientos and Llinás, unpublished observations).

Thalamic neurons also have complex intrinsic properties that allow them to function either as relay systems, or as oscillators and/or resonators. That these two modes are intrinsic to the cells and are controlled by their membrane potential has been studied both *in vitro* (Llinás and Jahnsen, 1982; Jahnsen and Llinás, 1984a,b; Hirsch et al., 1985; McCormick and Prince, 1986, 1987; Crunelli et al., 1987; Wilcox et al., 1988) and *in vivo* (Deschenes et al., 1984; cf. Steriade and Llinás, 1988) The basic electrophysiological phenomenology observed in these cells is shown in Figure 9.

From a slightly depolarized membrane potential, the outward current injection elicited a subthreshold depolarization (Figure 9A, second trace). When the current pulse was delivered from a more depolarized potential, regular, tonic firing was elicited as shown in the top trace of Figure 9A. Thus, at or near the resting potential, tonic firing is elicited by membrane depolarization. Accordingly, the response to an excitatory synaptic input would be a single excitatory postsynaptic potential (EPSP) that may trigger single spikes. A very different response was elicited when a similar current pulse was delivered from a hyperpolarized level as in Figure 9B. Under these conditions, the same outward current pulse showed in A, triggered an all-or-none burst of spikes. The uniformity of the waveform of the burst is demonstrated by the fact that several traces are superimposed in Figure 9B. The response comprises two distinct parts, a low-threshold spike (LTS), a slowly rising and falling triangular-like potential, and a rapid succession of fast spokes. As in the IO, the LTS is due to activation of a Ca^2+^ conductance that is deinactivated by membrane hyperpolarization. The amplitude of the low-threshold response is related to the membrane potential before its generation. This is shown in Figure 9C where a series of hyperpolarizing pulses of increasing amplitude was delivered from a slightly hyperpolarized membrane level.

The rate of rise and amplitude of the rebound response elicited at the current break increased with progressively larger hyperpolarizing pulses. At the two highest levels, the rebound potential reached the firing threshold for Na^+^ -dependent spikes. The deinactivation of the low-threshold Ca^2+^-dependent spike is also time-dependent; hyperpolarizing pulses of increasing duration produce graded deinactivation (Jahnsen and Llinás, 1984a), and complete recovery of the LTS occurs after a refractory period of 170–200 ms. Another characteristic of thalamic neurons, the presence of an A-like potassium current, may also be seen in Figure 9C as a longer time course to repolarization. Deinactivation of this conductance is responsible for the delay in the return of the potential to the holding potential at the end of the current injection.

A direct demonstration of the low threshold calcium conductance can best be described by the results from thalamic neuron voltage clamping. Such results are illustrated in Figures 9D,E. Indeed, the time course of the calcium inactivating T current is clearly demonstrable from a holding potential of 75 mV following Na and K conductance block by TTX and TEA respectively. The activation property of this current is shown in graph Figure 9E.

**Figure 10:**
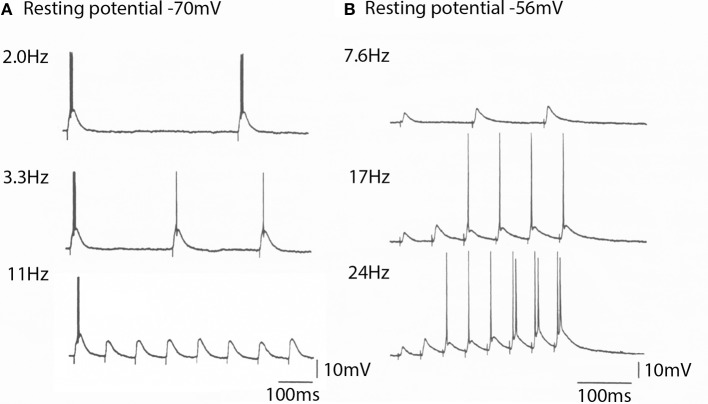
**The spike generation properties and EPSP amplitude generated by a thalamic neurons to cortico-thalamic volleys is membrane potential depend. (A)** At −70 mV the thalamic cell generated spikes at frequencies bellow 10 Hz. Note that the EPSPs generated are all of the same amplitude (bottom trace). **(B)** At a resting potential of −56 mV the EPSP amplitude for the same cortical volley was initially smaller, but increased in amplitude with stimulus frequency. It could follow high frequency stimulation and produce rapid neuronal spike firing. (From Pedroarena and Llinás, 2001.).

In addition to these voltage dependent conductances, thalamic neurons can modify their synaptic properties depending on membrane potential in a quite remarkable fashion. These properties are often not taken into account when considering their effects on arousal. Thus, fast reversible synaptic plasticity occurs in the thalamus by changes in postsynaptic membrane potential, independently of presynaptic volley size, and is rapidly reversible. It represents one of the few examples of rapid postsynaptically dependent synaptic plasticity as illustrated in Figure 10.

Three mechanisms are involved in this synaptic facilitation; (1) presynaptic short-term facilitation, (2) frequency–dependent activation of NMDA receptors, and (3) amplification of EPSP amplitude by intrinsic high-threshold conductances (Pedroarena and Llinás, 2001).

The significance of such finding resides in the fact that the thalamocortical system can quickly select functional states relating to gamma band allowing cognitive attractors to be continuously modulated by the combination of recurrent thalamocortical activity and the sensory input from the external world.

## Thalamic 40 Hz oscillations

**Figure 11:**
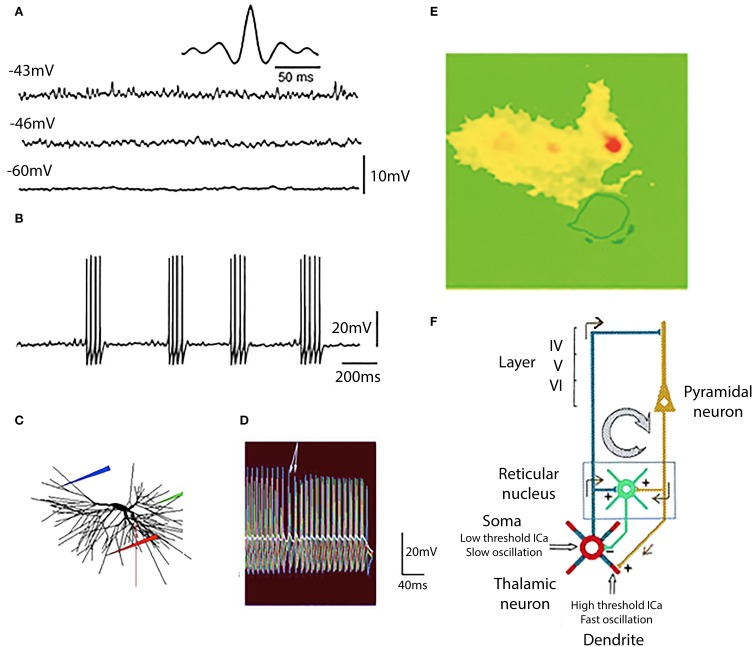
**Generation of gamma band oscillation by thalamic dendrites. (A)** Three different levels of membrane potential are accompanied by a rapid membrane potential oscillation with clear gamma band frequency at −46 and −43 mV. This is demonstrated by the dominant frequency at 37.5 Hz at −43 mV as shown in the auto-correlogram (insert). **(B)** At a membrane potential of −40 mV action potentials were generated at the peak of each oscillatory wavelet and so the subthreshold oscillatory membrane properties are transformed into gamma band spike frequency projected via thalamocortical axons on to the cortical mantle. **(C,D)** The mechanism for this gamma band oscillation was of dendritic origin was tested with a computer model. **(E)** Direct demonstration that the gamma oscillations are mostly dendritic and carried by calcium ions was accomplished using calcium specific fura 2 fluorescence imaging. The cell was depolarized to the level that elicited fast firing calcium entry was restricted to the dendritic tree (yellow and red) **(F)**. Diagram of the oscillatory properties of thalamic neurons and the recurrent inhibition at somatic level via the thalamic nucleus reticular nucleus. [**(A,B,E,F)** from Pedroarena and Llinás, 1997; **(C,D)** from Rhodes and Llinás, 2005.].

In addition to the now well-known thalamic currents responsible for the wake-sleep cycle (Steriade and Llinás, 1988), *in vitro* studies indicate that, in addition to the low frequency and alpha rhythms, a gamma band rhythm is also present in thalamic neurons. This is particularly clear at dendritic levels and is supported by P/Q type calcium channels (Pedroarena and Llinás, 1997) and is essential in the generation of cognitive functions (Llinás et al., 2007). Indeed depolarization by direct current injection elicits well defined high frequency at potentials of −46 and −43 mV (Figure 11A) and the oscillations that can reach threshold for spike initiation at −40 mV (Figure 11B). These high frequency oscillations were blocked by P/Q channel blocker SFtx (Llinás et al., 1989; Mintz et al., 1992; cf Nimmrich and Gross, 2012).

The relationship of dendritic spikes and gamma oscillations was examined in a mathematical model of thalamocortical relay cells (Rhodes and Llinás, 2005). The model incorporated the generation of somatic spikes, low threshold rebound spike bursts, and fast somatic oscillations near threshold. In the distal dendrites the model neuron generated both isolated high-threshold calcium spikes and low threshold calcium spikes that did not require a high dendritic density of calcium channels. Somatic depolarization elicited firing in a dendrite (Figure 11C, red electrode; Figure 11D, red traces) that leads to subthreshold oscillations in the some (Figure 11D, white trace). When somatic depolarization ended, arrows in Figure 11C, dendritic spiking stopped. A similar pattern was seen when the location of the dendritic electrode was moved (blue and green in Figures 11C,D). The period of firing in the distal dendrites controlled the somatic oscillation frequency.

The gamma oscillations are generated in the dendrites as shown by dye imaging studies such as shown in Figure 11E where fluorescence of the calcium-specific dye flura 2 is restricted to the dendrites (Pedroarena and Llinás, 1997).

In thalamo-cortical slices, where the reciprocal connectivity is intact, thalamic stimulation results in the recurrent activity of the thalamocortical loop (Figure 11F). It is interesting to note that the high frequency cortical return excitation is mostly restricted to the dendritic thalamic compartment (Figure 11E). From the above it has been concluded that this dendritic conductance are not only related to oscillatory gamma band activity, but are essential in the generation of brain gamma band activity and of cognitive functions, as was demonstrated in mice genetically modified to delete P/Q type channels (Cav 2.1 null mice) (Llinás et al., 2007).

**Figure 12:**
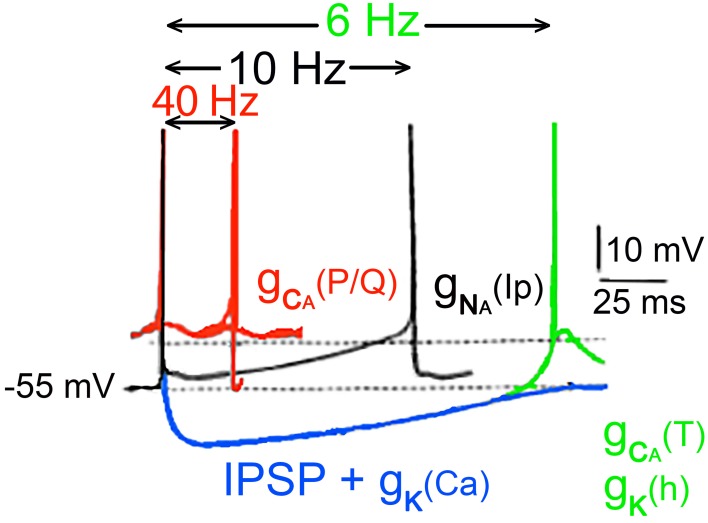
**Conducances underlying the oscillatory properties of thalamic neurons**. In black, usual Na^+^ dependent spike is followed by an after hyperpolarization generated by the classical voltage-sensitive K^+^ conductance. Depending on membrane potential, this event can be followed by a persistent sodium current [(g_Na(Ip)_) black spikes]. Following an inhibitory synaptic potential a hyperpolarization dependant deinactivation of type a g_Ca (T)_ conductance, and the simultaneous inactivation of the potassium conductance g_K(Ih)_ together generate a rebound response (green). The membrane potential is brought back to the threshold for the fast spike by the slow potassium conductance. In addition to the 10-Hz oscillations, slower oscillations (about 6 Hz) can occur by the rebound excitation (blue trace) following hyperpolarization of the cell [g_K_ (Ca)] and inhibitory postsynaptic potentials (IPSPs). Such hyperpolarization deinactivates the low-threshold Ca^2+^ conductance generating a rebound low threshold spike, which triggers the process once again activating potassium conductance (Ih). (Jahnsen and Llinás, 1984b,c; McCormick and Prince, 1986).

In all then, six ionic conductances have been described in thalamic neurons, in addition to those that underlie the axosomatic action potential as summarized in Figure 12.

1. A voltage-dependent persistent, or very slowly inactivating somatic, Na^+^ conductance, [g_Na(Ip)_] This conductance generates a slow rebound depolarization in thalamic cells and plays a role in the genesis of the 10-Hz oscillation Figure 10 (Jahnsen and Llinás, 1984b).
2. A Ca^2+^-dependent potassium somatic conductance, [**g**_K(Ca)_] which underlies the AHP. This conductance was demonstrated *in vitro* by the marked reduction of the AHP, after application of Ca^2+^ channel blockers (Jahnsen and Llinás, 1984b). The AHP amplitude is about 12 mV and can be reversed by inward current injection (Deschenes et al., 1984; Jahnsen and Llinás, 1984b).
3. A fast, transient somatic potassium conductance (**g**_IA_), responsible for the slow return to baseline following hyperpolarization (Jahnsen and Llinás, 1984b; Kita and Kitai, 1986), which can prevent the abrupt recovery of neurons from a hyperpolarized condition. In fact, the duration of the hyperpolarization that generates the rebound response is aided by the presence of the transient K+ current.
4. A low-threshold, rebound, somatic Ca^2+^ conductance [g_Ca(T)_]. This conductance is inactive at the resting potential and deinactivates with hyperpolarization.
5. A high-threshold, dendritic Ca^2+^ conductance [G_Ca(P/Q)_]. This conductance triggers all-or-none depolarizing responses followed by activation of a Ca-gated K conductance.
6. A somatic h-type potassium channel.

In contrast to the IO, the high threshold Ca^2+^ conductance is not strong in thalamic cells. Because of this difference, the dendritic Ca^2+^-dependent spike does not dominate the firing of the thalamic neuron, allowing it a wider range of firing properties that that in IO neurons. The amplitude of the dendritic Ca^2+^ channels current in thalamic dendrites is smaller than that in inferior olivary neuron.

The six conductances combine to give thalamic neurons their unique oscillatory properties, as diagramed in Figure 12. At membrane potentials positive to −55 mV, fast action potentials are generated (red traces). At membrane levels near −55 mV, two types of firing are seen (black traces). In one case, the fast sodium-dependent spike is followed by an AHP, due to an increase in both the classical voltage-activated potassium conductance (g_K_) and by a Ca^2+^-activated K^+^ conductance [g_K(Ca)_] that generates an AHP lasting for 70 ms or so, allowing the cell to fire at a frequency near 10 Hz. The response can be further augmented as a rebound from the inhibitory postsynaptic potential (IPSP) in blue.

Thalamic cell firing is basically produced by a slow depolarization of the cell produced by the activation of the persistent Na+ conductance [g_Na(IP)_], which can serve as a continuous depolarizing drive once it is activated. Once the [g_Na(IP)_] takes over it depolarizes the cell until another spike is generated and the process repeats itself, with a 10-Hz rhythmicity. If, on the other hand, the hyperpolarizing potassium conductances are combined with an A potassium current and/or IPSPs, the neurons are hyperpolarized sufficiently to deinactivate the low-threshold Ca^2+^ conductance [g_Na(Ip)_] and to inactivate a potassium conductance (Ih) resulting in an oscillatory responses at frequencies near 6 Hz. Thus, their intrinsic properties allow thalamic neurons to display a versatility whereby they switch between tonic and phasic responses as diagrammed in Figure 12.

The point to be emphasized here is not the difference between these two groups of cells but rather the fact that they both have intrinsic properties that give them distinctive firing characteristics. From the above, it follows that the nervous system is constantly in action and that the patterns of activity arising from the sensory inputs and from the corollary discharge of motor outputs, are but a small modulatory component of the overall activity of the brain. Beyond these conductances, the thalamic neuron oscillatory patterns can also be generated via synaptic activation as elegantly demonstrated *in vitro* studies by Sohal et al. (2006).

## Cortical neurons

The electrophysiology of cortical neurons has been extensively studied (Yuste et al., 2005) and the morphology-related intrinsic firing patterns in simulated neocortical pyramidal cells has been examined as well (Korogod and Tyc-Dumont, 2009). In this summary I will touch briefly on neuronal aspects of cortical neurons that relate very specifically to 40 Hz activation in relation to the intrinsic properties of a particular type of interneuron, the sparsely spinous neurons of the fourth cortical layer.

From an *in vitro* point of view, our research in the cerebral cortex of the guinea pig points to the existence of neurons in the fourth layer that have intrinsic subthreshold electroresponsive properties that endow these cells with a 30- to 45-Hz membrane potential oscillation (Llinás et al., 1991). These cells, which are often silent after penetration, demonstrate oscillation on direct membrane depolarization. On occasion, the cells may also show spontaneous oscillations at that frequency. When this occurs, further depolarization produced by direct current injection will generate a spike at the peak of the depolarizing phase of each oscillation. In other recordings in similar neurons, it was also found that a voltage-dependent persistent sodium conductance may underlie the generation of 40-Hz oscillation, which, in that case, outlasts the duration of the depolarizing pulse.

**Figure 13:**
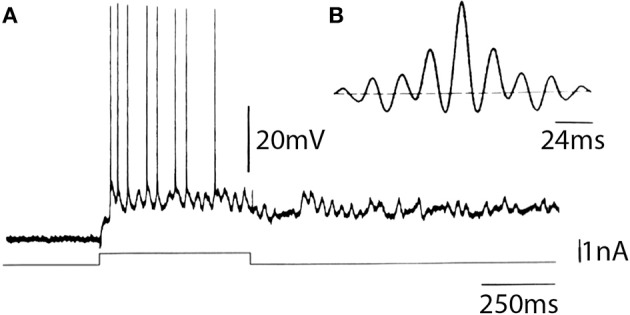
***In vitro* intracellular recording from a sparsely spinous neuron of the fourth layer of the frontal cortex of guinea pig. (A)** Characteristic response obtained in the cell following direct depolarization, consisting of sustained subthreshold oscillatory activity on which single spikes can be observed. **(B)** Autocorrelogram of the intrinsic oscillatory frequency indicated a 42 Hz intrinsic oscillation (Llinás et al., 1991).

Examples of such recordings are shown in Figure 13A. Autocorrelation analysis of this response (Figure 13B) demonstrates that the frequency of oscillation of this cell was 42-Hz.

Following intracellular staining, these fourth-layer neurons were recognized as the sparsely spinous neurons that have been described by anatomists as being GABAergic and as having axons that ascend to the third layer and descend to the fifth layer in the cortex (Peters and Saint-Maie, 1984).

As opposed to the oscillations observed in the IO and thalamus, the oscillation of these fourth-layer inhibitory interneurons appears to be generated by a voltage-dependent sodium conductance followed by a potassium conductance, probably of the voltage- or Na^+^-dependent variety. The sodium dependence of this oscillation was demonstrated by the addition of TTX to the bath, which blocked the oscillations generated by direct stimulation as well as those spontaneously generated. The subthreshold oscillations were, in fact, the first to disappear, followed by the blockage of the all-or-none fast spikes.

Other examples of similar types of oscillations, but with a lower frequency, have been observed in the giant stellate cells of the entorhinal cortex (Alonso and Llinás, 1992) as well as in the neurons of the nucleus parabrachialis in the brainstem (Leonard and Llinás, 1990). In contrast to the 40-Hz oscillations displayed by the cortical neurons, the latter two cell types oscillate at a frequency of 6–12 Hz, i.e., in the range of the theta and low alpha rhythms. The point of interest here is that similar current may generate different oscillatory frequencies, depending on the kinetics of the voltage-dependent conductances. Equally significant is the fact that oscillations at a similar frequency may have different ionic bases in different types of cells. This is probably related to secondary events that oscillation may ultimately regulate. Thus, as some cell oscillations may be a form of communication, in others, the hippocampus for example, oscillation may serve to trigger secondary changes such as long-term potentiation (Larson and Lynch, 1986). These latter require the activation of Ca^2+^-dependent second messengers, in which case Ca^2+^ -based oscillation would be a significant parameter (Llinás and Steriade, 2006).

*In vivo* cortical studies that have taken a broader perspective than that provided by single cell *in vitro* electrophysiology have demonstrated in the cat, that high-frequency activity occurs in motor areas 4y, 6aB and in the posterior parietal associative area 5a during motionless focused attention (Bouyer et al., 1987). These recordings were obtained from the surface of the cortex, as well as from depth field analyses. With respect to the visual cortex, it was demonstrated (Gray et al., 1989) that, following specific visual input, a 40-Hz oscillation may be observed as a field potential envelope, and as single units in the overall envelope. This 40-Hz oscillation appears to be present only when the given cortical area is activated by an optimal physiological stimulus.

The question of the role of the intrinsic electrical properties of neurons in the overall function of the CNS must be defined, then, at the cell ensemble level. Perhaps one of the most interesting issues concerning global brain function relates to the rediscovery in recent years of 40-Hz oscillation, which may be observed in the cortex under certain conditions. In fact, 40-Hz oscillations have been observed in the cortex under certain conditions. For example, there are 40-Hz oscillations during physiological stimulation of the visual (Gray et al., 1989) or auditory cortex (Galambos et al., 1981; Spydell et al., 1985; Mäkelä and Hari, 1987; Johnson et al., 1988). In its absence, as occurs when P type calcium channels are (Ca 3.1) are deleted, there is a total lack of cognitive function. Such oscillations have also been recorded during intensive attention states such as occur when a predator is stalking its prey (Bouyer et al., 1987), or in humans, during the state of elevated attention prior to the execution of complicated tasks (Sheer, 1984). Similar activity as measured from the scalp by electric and magnetic means in humans appears to be well correlated with cognitive tasks and seems to be altered under pathological conditions (Ribary et al., 1989, 1991).

**Figure 14:**
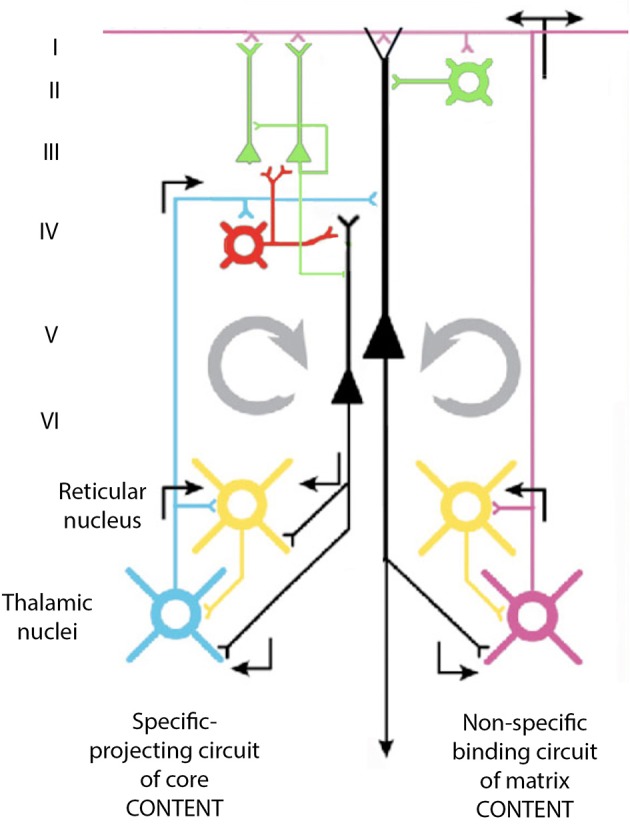
**Diagram of the proposed cortico-thalamo-cortical reverberating circuit, which may underlie 40-Hz oscillation at the cortex**. See text.

The issue here is that the oscillatory events may be used in intercellular communication as well as in the modulation of the intracellular milieu. From the point of view of 40-Hz oscillations, the oscillation in the GABAergic neurons may, in fact, be the origin of the macroscopic 40-Hz observed in the cortex. Indeed, since these cells receive direct synaptic input from thalamic neurons, and since they relay inhibition to pyramidal cells at a 40-Hz frequency, this will, in turn, produce a resonance activation of the pyramidal cells at a frequency of 40 Hz. The question of interest, then, is the mechanism by which such oscillations may become sufficiently synchronous to generate macroscopic events. Our own view on this matter is illustrated in Figure 14.

Thalamic input via projection neurons reaches the neurons of the fourth layer of the cortex that generate 40-Hz oscillation. These cells, which are GABAergic, can generate a 40-Hz IPSP on pyramidal neurons and allow them to fire at 40-Hz as a rebound from abrupt inhibition. Pyramidal cells, in turn, activate, via collaterals, the thalamic projection neurons as well as the neurons of the reticular thalamus and the interneurons at the thalamus itself. The direct excitatory input at 40-Hz to the projection thalamic cells, as well as their disynaptic inhibition via the reticularis and intrathalamic GABAergic neurons, contributes to the thalamic oscillations at 40-Hz. This thalamic oscillation is then signaled back to the cortex, establishing a large resonant oscillation between the thalamus and the cortex, which can recruit sufficient elements to generate the synchronicity observed at both intracellular and extracellular levels in the cortex and thalamus.

According to this view, the inhibitory neurons that contact pyramidal cells directly may force them in combination to a synchronous excitatory input to rebound oscillation. These cells will then generate, via their descending axons, 40-Hz excitation of cells in the nucleus reticularis thalami (NRT), the intrinsic inhibitory neurons in the thalamus, and the projection thalamic neurons themselves.

Since the NRT cells are inhibitory (cf. Steriade and Llinás, 1988) they would further increase the resonance property of the thalamocortical system by their own driven 40-Hz input back through the fourth-layer interneurons. In this manner, a cortico-thalamo-cortical resonance even may actually be the basis of this 40-Hz rhythm recorded at the cortical level.

While Gray et al. (1989) have not recorded oscillation in the thalamus at 40-Hz, other investigators (Fuster et al., 1965) had, in fact, published intracellular recordings from geniculate neurons following light stimulation demonstrating clear 40-Hz activity. This indicates that activity at such frequencies is not a cortical phenomenon exclusively but may also be seen at the thalamic level.

**Figure 15:**
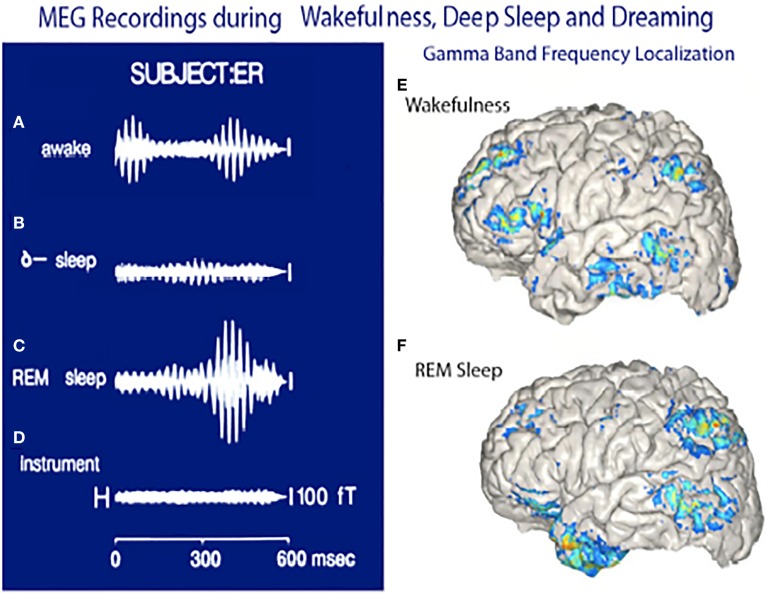
**Magnetoencephalographic (MEG) recordings in three functional states. (A)** Magnetic recording demonstrating gamma band activity following a sensory stimulus in awake subject. **(B)** Recordings from same subject during deep, dreamless sleep. **(C)** Gamma band activity while dreaming. **(D)** Instrument noise in the absence of a subject. **(E)** Localization of gamma band activity in an awake subject note frontal and parietal and temporal association lobe activity. **(F)** Localization of gamma band activity recorded when the subject was dreaming. Note the lack of frontal lobe activity and the powerful activation of the temporal pole. (Llinás and Ribary, 1993 and unpublished observations.).

The MEG recordings shown in Figure 15 suggest that mostly somatosensory and visual auditory association cortices are active as well as the anterior temporal pole (amygdala) are the main players during dreaming while the frontal lobe remains only sparsely active (Figure 15F). Similar findings have been reported using MEG by Ioannides et al. (2004).

The functional significant of 40-Hz oscillation in the thalamocortical system becomes particularly interesting when one considers that it may serve as the basis for the temporal correlation of events that must be considered as a single perceptual or motor entity, the so called conjunction principle. This temporal superposition, in fact, may be at the very core of global brain function. Indeed, it may actually be observed using magnetic recording in humans, where 40-Hz activity can be demonstrated to be organized quite widely, demonstrating a phase shift from the front of the brain onto the back with a sweep speed in the rostro-caudal direction of about 5–10 ms (Ribary et al., 1989, 1991). This phase shift suggests that, via the activation of the NRT, a conjunctional type of activity may be generated at the thalamus, which allows thalamocortical resonance in a global, organized manner. This, in its most simplified form, could serve to scan the brain front to back 200 times a second. Such scanning would be viewed as the basis for the generation of unitary perceptual entities out of many sensory and motor vector components, which represent the details of the perceived world.

Ultimately, we are then faced with a system that addresses the external world, not as a slumbering machine to be awoken by the entry of sensory information, but rather as a continuously humming brain willing to internalize and incorporate into its intimate activity an image of the external world, but always in the context of its own existence and its own intrinsic electrical activity. Most fundamental, however, is the fact that the system is not merely a computational entity, but rather that issues are substrate dependent (neurons) and, moreover, that ionic events such as the presence of P/Q calcium channel activity are actually crucial to its ultimate function. From a more global perspective it is the dialog between the incoming information arising, in mammals, from the dorsal thalamus that provides the content in our everyday cognitive activity, and the nonspecific system that provides context (i.e., the attention), that we give to such inputs (Llinás et al., 1994).

### Conflict of interest statement

The author declares that the research was conducted in the absence of any commercial or financial relationships that could be construed as a potential conflict of interest.
